# Prognostic significance of surgery and radiotherapy in elderly patients with localized prostate cancer: establishing and time-based external validation a nomogram from SEER-based study

**DOI:** 10.1186/s12894-023-01384-6

**Published:** 2024-01-06

**Authors:** Chenghao Zhanghuang, Jianjun Zhu, Ye Li, Jinkui Wang, Jing Ma, Li Li, Zhigang Yao, Fengming Ji, Chengchuang Wu, Haoyu Tang, Yucheng Xie, Bing Yan, Zhen Yang

**Affiliations:** 1 Department of Urology, Kunming Children’s Hospital (Children’s Hospital affiliated to Kunming Medical University), Kunming, People’s Republic of China; 2 Department of Oncology; Yunnan Children solid Tumor Treatment Center, Kunming Children’s Hospital (Children’s Hospital affiliated to Kunming Medical University), Kunming Children’s Solid Tumor Diagnosis and Treatment Center, Kunming, People’s Republic of China; 3 Yunnan Key Laboratory of Children’s Major Disease Research, Kunming Children’s Hospital (Children’s Hospital affiliated to Kunming Medical University); Yunnan Province Clinical Research Center for Children’s Health and Disease, Kunming Children’s Solid Tumor Diagnosis and Treatment Center, Yunnan Clinical Medical Center for Pediatric Diseases, Kunming, People’s Republic of China; 4 Chongqing Key Laboratory of Children Urogenital Development and Tissue Engineering; Chongqing Key Laboratory of Pediatrics; Ministry of Education Key Laboratory of Child Development and Disorders; National Clinical Research Center for Child Health and Disorders; China International Science and Technology Cooperation base of Child development and Critical Disorders; Department of Urology, Children’s Hospital of Chongqing Medical University, Chongqing, 400015 China; 5 Department of Otolaryngology, Kunming Children’s Hospital (Children’s Hospital affiliated to Kunming Medical University), Kunming, People’s Republic of China; 6 Department of Pathology, Kunming Children’s Hospital (Children’s Hospital affiliated to Kunming Medical University), Kunming, People’s Republic of China

**Keywords:** Nomogram, SEER, Elderly, Localized, Prostate cancer, CSS, OS

## Abstract

**Objective:**

Prostate cancer (PC) is a significant disease affecting men’s health worldwide. More than 60% of patients over 65 years old and more than 80% are diagnosed with localized PC. The current choice of treatment modalities for localized PC and whether overtreatment is controversial. Therefore, we wanted to construct a nomogram to predict the risk factors associated with cancer-specific survival (CSS) and overall survival (OS) in elderly patients with localized PC while assessing the survival differences in surgery and radiotherapy for elderly patients with localized PC.

**Methods:**

Data of patients with localized PC over 65 years were obtained from the Surveillance, Epidemiology, and End Results (SEER) database. Univariate and multivariate Cox regression models were used to determine independent risk factors for CSS and OS. Nomograms predicting CSS and OS were built using multivariate Cox regression models. The consistency index (C-index), the area under the subject operating characteristic curve (AUC), and the calibration curve were used to test the accuracy and discrimination of the prediction model. Decision curve analysis (DCA) was used to test the potential clinical value of this model.

**Results:**

A total of 90,434 patients over 65 years and diagnosed with localized PC from 2010 to 2018 were included in the study. All patients were randomly assigned to the training set (*n* = 63,328) and the validation set (*n* = 27,106). Univariate and multivariate Cox regression model analysis showed that age, race, marriage, T stage, surgical, radiotherapy, prostate-specific antigen (PSA), and Gleason score (GS) were independent risk factors for predicting CSS in elderly patients with localized PC. Age, race, marriage, surgery, radiotherapy, PSA, and GS were independent risk factors for predicting OS in elderly patients with localized PC. The c-index of the training and validation sets for the predicted CSS is 0.802(95%CI:0.788–0.816) and 0.798(95%CI:0.776–0.820, respectively). The c-index of the training and validation sets for predicting OS is 0.712(95%:0.704–0.720) and 0.724(95%:0.714–0.734). It shows that the nomograms have excellent discriminatory ability. The AUC and the calibration curves also show good accuracy and discriminability.

**Conclusion:**

We have developed new nomograms to predict CSS and OS in elderly patients with localized PC. After internal validation and external temporal validation with reasonable accuracy, reliability and potential clinical value, the model can be used for clinically assisted decision-making.

**Supplementary Information:**

The online version contains supplementary material available at 10.1186/s12894-023-01384-6.

## Background

Prostate cancer (PC) is a significant disease that affects men’s health worldwide. It is the second most common form of cancer in men, after non-melanoma skin cancer, such as basal cell carcinoma and squamous cell carcinoma [1]. In 2022, new cases of PC in the United States were expected to reach 268,490, while in 2022, more than 34,500 deaths are expected to occur from PC in the United States, or about 11% of male cancer deaths [2]. PC is a senile disease, and its onset is closely related to age. However, the age of diagnosis has advanced with the popularity of PSA screening, with more than 60% of patients over 65 [3]. More than 80% of these were diagnosed with localized PC [4]. However, older patients were not eligible for enrollment in large randomized trials, and existing guidelines for the management of patients with prostate cancer do not provide specific treatment recommendations for older men [5]. Therefore, it is urgent to provide a clinical treatment basis for elderly patients with localized prostate cancer.

Localized PC refers to a PC with a T stage of T2c and below [6]. According to the current treatment protocol, most patients have a better prognosis. However, PC itself is a highly heterogeneous group of diseases, and patients’ disease progression and prognosis vary greatly. Moreover, the mortality rate of elderly patients is still higher due to comorbidity [7]. Therefore, finding the risk factors for the prognosis and localized PC in old age is essential. Second, current treatment modalities for localized PC include active surveillance, surgical treatment, and radiotherapy [8]. In addition, surgical treatment is considered to be the gold standard for high-risk PC [9]. However, the current choice of treatment modalities for localized PC and whether overtreatment is currently controversial. Therefore, we wanted to construct a predictive model to evaluate further the survival differences in surgical approach and radiotherapy for older patients with localized PC.

Current nomograms developed based on the SEER database have been widely used to predict multiple tumours accurately. Most of the nomograms for PC were concentrated in metastasis patients, especially bone metastasis patients or patients with characteristic GS scores [10–12]. However, researchers have yet to develop relevant predictive models for the large group of elderly-limited PCs. Due to comorbidity, its OS cannot respond to cancer-specific deaths from cancer. Therefore, the purpose of this study is to use SEER database data to develop new nomograms to predict independent risk factors related to OS and CSS in elderly localized PC patients and evaluate the survival differences between surgery and radiotherapy to provide auxiliary decision-making for clinicians and patients.

## Patients and methods

### Data source and data extraction

Information for all patients was extracted from the SEER database, and our target population was patients aged 65 years and older diagnosed with localized PC between 2010 and 2018. The SEER database is a national cancer database containing data from 18 cancer registries, covering approximately 30% of the population. Since the data in the SEER database is publicly available and the patient information is hidden. Ethical approval and patient informed consent was therefore not required. This study followed the research guidelines published in the SEER database.

**Fig. 1:**
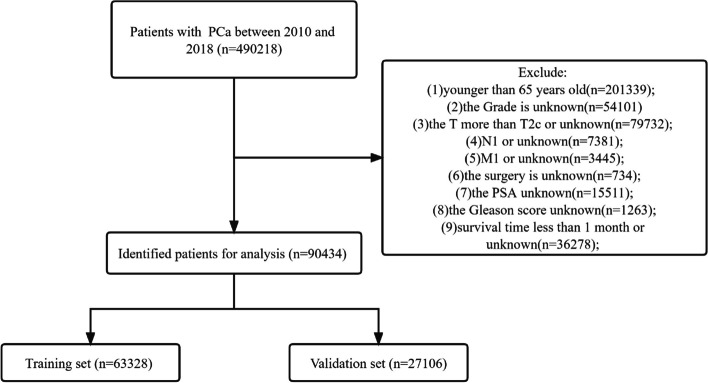
Flowchart for inclusion and exclusion of elderly patients with localized prostate cancer

We collected clinicopathological information for all elderly patients with localized PC as well as patient follow-up results, including age, race, year of diagnosis, marital status, histological tumour grade, T stage, surgery, radiotherapy, chemotherapy, PSA, GS, survival status, cause of death, and survival time. According to the official description given in the SEER database, the Grade classification is divided into Grade I: well-differentiated; Grade II: moderately differentiated; Grade III: poorly differentiated; and Grade IV: undifferentiated or anaplastic. The inclusion criteria for this study were: (1) patients aged≥65 years; (2) pathological diagnosis of PC; (3) TNM stage was T1NOMO, T2N0M0. Exclusion criteria: (1) patients younger than 65 years old; (2) tumour grade unknown; (3) T stage above T2 or specific T stage unknown; (4) N1 or N stage unknown; (5) M1 or M stage unknown; (6) unknown surgical method; (7) PSA unknown; (8) GS unknown; (9) survival time less than 1 month or unknown. The flowchart of patient inclusion and exclusion is shown in Fig. 1.

### Development and validation of the nomograms

We first randomized the patients into the training set (70%) and the validation set (30%) for nomogram development and internal validation. Furthermore, we performed external temporal validation using data from PC patients from 2016 to 2018. Univariate and multivariate Cox proportional regression models were used to identify independent risk factors that affected patient CSS and OS in the training set. New nomograms were constructed based on multiple Cox regression models and were used to predict the CSS and OS at 3,5 and 8 years, respectively. The accuracy of the nomogram was verified using a calibration curve of 1000 bootstrap samples. The consistency index (c-index) and the area under the subject operating characteristic curve (AUC) were used to test the accuracy and discrimination of the model.

### Clinical application

Decision analysis curves (DCA) were used to evaluate the nomogram’s clinical value and compare it with the TNM staging system and the D’Amico risk stratification system. In addition, we also calculated the risk for each patient from the nomogram. All patients were divided into high-risk and low-risk groups based on the cutoff value of the subject’s working characteristic curve (ROC). The Log-rank test and the Kaplan-Meier (K-M) curve examined CSS and OS at 3,5,8 years in high-risk and low-risk patients. In addition, we evaluated the CSS and OS differences in different surgical and radiotherapy methods between patients in the high-risk and low-risk groups.

### Statistical analysis

Continuous variables such as age were tested for normal distribution, which can be described by mean and standard deviation, and categorical variables (race, marriage, tumour grade, T stage, surgery, radiotherapy, chemotherapy, PSA, and GS) were described by frequency (%). Chi-square or non-parametric U tests were used for comparison between groups. The Cox regression model analyzed patient prognostic factors, and the log-rank test and K-M curves analyzed patient survival differences. Statistical analysis was performed using R software version 4.1.0 and SPSS26.0.The R package includes “Survival”, “ggDCA”, “DynNom”, and “RMS”.*P* values less than 0.05 were considered statistically significant.

## Results

### Clinical features

A total of 90,434 elderly patients diagnosed with localized PC between 2010 and 2018 were included in this study. It was randomly assigned to the training set (*n* = 63,328) and the validation set (*n* = 27,106). The mean age of the training set and validation set was 71.4 ± 5.28 years and 71.4 ± 5.33 years, respectively. Our patients were predominantly white according to race (78.3%) and married according to marital status (67.6%). Tumours included grade I (18.97%), grade II (44.73%), grade III (36.27%), and grade IV (0.03%). All the T stages were mainly T1c (60.8%). Patients receiving non-surgical treatment (73.2%), patients undergoing local tumour resection (5.8%), and patients undergoing radical prostatectomy (21.0%). Only 0.2% of the patients received chemotherapy. 34.0% of the patients received Beam radiation, 6.1% received Radioactive implants or isotopes, and 4.4% received Combination radiotherapy, while 55.5% did not. The detection rates of GS 1–6, GS 7 and GS 8–10 were 36.6, 44.5 and 18.8%, respectively. PSA<10 ng/ml(71.9%), 10 ≤ PSA<20 ng/ml(19.4%), PSA ≥ 20 ng/ml(8.7%)。There was no significant statistical bias in the data of both groups, and the results are shown in Table 1.

**Table 1:** Clinicopathological characteristics of elderly patients with PC

	All	Training cohort	Validation cohort	p
	*N* = 90,434	*N* = 63,328	*N* = 27,106	
Age	71.4 (5.29)	71.4 (5.28)	71.4 (5.33)	0.765
Race:				0.460
white	70,848 (78.3%)	49,670 (78.4%)	21,178 (78.1%)	
black	11,557 (12.8%)	8082 (12.8%)	3475 (12.8%)	
other	8029 (8.9%)	5576 (8.8%)	2453 (9.1%)	
Marital:				0.150
No	29,277 (32.4%)	20,595 (32.5%)	8682 (32.0%)	
Married	61,157 (67.6%)	42,733 (67.5%)	18,424 (68.0%)	
Grade:				0.096
I	17,153 (18.97%)	11,990 (18.93%)	5163 (19.05%)	
II	40,450 (44.73%)	28,459 (44.94%)	11,991 (44.25%)	
III	32,798 (36.27%)	22,852 (36.09%)	9946 (36.69%)	
IV	33 (0.03%)	27 (0.04%)	6 (0.01%)	
T:				0.995
T1a-b	2900 (3.2%)	2036 (3.2%)	864 (3.2%)	
T1c	54,981 (60.8%)	38,488 (60.8%)	16,493 (60.8%)	
T2a-b	13,055 (14.4%)	9145 (14.4%)	3910 (14.5%)	
T2c	19,498 (21.6%)	13,659 (21.6%)	5839 (21.5%)	
Surgery:				0.410
No	66,237 (73.2%)	46,465 (73.4%)	19,772 (72.9%)	
Local tumor resection	5209 (5.8%)	3631 (5.7%)	1578 (5.9%)	
Radical prostatectomy	18,988 (21.0%)	13,232 (20.9%)	5756 (21.2%)	
Chemotherapy:				0.611
No	90,262 (99.8%)	63,204 (99.8%)	27,058 (99.8%)	
Yes	172 (0.2%)	124 (0.2%)	48 (0.2%)	
Radiation:				0.840
No	50,159 (55.5%)	35,089 (55.4%)	15,070 (55.6%)	
Beam radiation	30,763 (34.0%)	21,596 (34.1%)	9167 (33.8%)	
Radioactive implants or isotopes	5549 (6.1%)	3868 (6.1%)	1681 (6.2%)	
Combination	3963 (4.4%)	2775 (4.4%)	1188 (4.4%)	
Gleason:				0.548
≤ 6	33,137 (36.6%)	23,195 (36.6%)	9942 (36.7%)	
7	40,277 (44.5%)	28,266 (44.6%)	12,011 (44.3%)	
≥ 8	17,020 (18.9%)	11,867 (18.8%)	5153 (19.0%)	
PSA:				0.369
<10	64,984 (71.9%)	45,455 (71.8%)	19,529 (72.0%)	
10–20	17,569 (19.4%)	12,377 (19.5%)	5192 (19.2%)	
>20	7881 (8.7%)	5496 (8.7%)	2385 (8.8%)	
CSS:				0.194
Dead	1911 (2.1%)	1312 (2.1%)	599 (2.2%)	
Alive	88,523 (97.9%)	62,016 (97.9%)	26,507 (97.8%)	
OS:				0.871
Dead	9584 (10.6%)	6704 (10.6%)	2880 (10.6%)	
Alive	80,850 (89.4%)	56,624 (89.4%)	24,226 (89.4%)	
Survival.months	48.7 (29.1)	48.6 (29.1)	48.9 (29.2)	0.194

### COX regression analysis

The univariate Cox regression model was first used to analyze and screen for factors associated with patient survival in the training set. The results showed that age, race, marriage, tumour grade, T stage, surgery, chemotherapy, radiotherapy, PSA, and biopsy GS were all factors associated with patient outcome. Then, a multivariate Cox regression model was used to screen for independent risk factors associated with CSS and OS in older patients with PC localized PC. The results showed that age, race, marriage, T stage, surgery, radiotherapy, PSA, and GS were independent risk factors affecting patients’ CSS. In contrast, age, race, marriage, surgery, radiotherapy, PSA, and GS were independent risk factors affecting patients’ OS. Analysis results are shown in Tables 2 and 3, respectively.

**Table 2:** Univariate and multivariate analyses of CSS in training cohort

	Univariate			Multivariate		
	HR	95%CI	P	HR	95%CI	P
Age	1.14	1.13–1.14	< 0.001	1.057	1.048–1.067	< 0.001
Race						
white						
black	1.33	1.14–1.54	< 0.001	1.149	0.986–1.339	0.075
other	0.76	0.62–0.93	0.009	0.616	0.5–0.76	< 0.001
Marital						
No						
Married	0.6	0.54–0.67	< 0.001	0.768	0.688–0.857	< 0.001
Grade						
I						
II	1.39	1.05–1.84	0.023			
III	3.99	3.05–5.22	< 0.001			
IV	6.01	1.47–24.62	0.013			
T						
T1a-b						
T1c	0.35	0.28–0.42	< 0.001	0.722	0.541–0.964	0.027
T2a-b	0.31	0.25–0.4	< 0.001	0.767	0.558–1.053	0.101
T2c	0.21	0.17–0.26	< 0.001	1.087	0.783–1.507	0.619
Surgery						
No						
Local tumor excision	2.45	2.11–2.86	< 0.001	1.39	1.111–1.737	0.004
Radical prostatectomy	0.23	0.18–0.29	< 0.001	0.177	0.134–0.235	< 0.001
Chemotherapy						
No						
Yes	2.96	1.41–6.22	0.004			
Radiation						
No						
Beam radiation	0.95	0.85–1.07	0.374	0.468	0.412–0.531	< 0.001
Radioactive implants or isotopes	0.46	0.35–0.62	< 0.001	0.458	0.341–0.616	< 0.001
Combination	0.84	0.64–1.1	0.204	0.422	0.319–0.557	< 0.001
PSA						
<10						
10–20	2.27	1.99–2.59	< 0.001	1.455	1.27–1.668	< 0.001
>20	6.8	6–7.72	< 0.001	2.431	2.111–2.8	< 0.001
Gleason						
≤ 6						
7	1.95	1.66–2.29	< 0.001	2.158	1.824–2.553	< 0.001
≥ 8	8.19	7.04–9.53	< 0.001	6.191	5.217–7.348	< 0.001

**Table 3:** Univariate and multivariate analyses of OS in training cohort

	Univariate			Multivariate		
	HR	95%CI	P	HR	95%CI	P
Age	1.11	1.11–1.11	< 0.001	1.072	1.067–1.076	< 0.001
Race						
white						
black	1.35	1.27–1.45	< 0.001	1.289	1.204–1.38	< 0.001
other	0.73	0.66–0.8	< 0.001	0.654	0.595–0.72	< 0.001
Marital						
No						
Married	0.64	0.61–0.67	< 0.001	0.753	0.716–0.791	< 0.001
Grade						
I						
II	1.09	0.99–1.2	0.081			
III	1.73	1.57–1.9	< 0.001			
IV	1.26	0.47–3.38	0.642			
T						
T1a-b						
T1c	0.43	0.39–0.48	< 0.001			
T2a-b	0.38	0.34–0.43	< 0.001			
T2c	0.24	0.22–0.27	< 0.001			
Surgery						
No						
Local tumor excision	1.85	1.72–2	< 0.001	1.108	0.982–1.25	0.097
Radical prostatectomy	0.35	0.32–0.38	< 0.001	0.35	0.31–0.395	< 0.001
Chemotherapy						
No						
Yes	1.79	1.17–2.75	0.007			
Radiation						
No						
Beam radiation	1.09	1.04–1.15	0.001	0.691	0.652–0.733	< 0.001
Radioactive implants or isotopes	0.72	0.64–0.8	< 0.001	0.658	0.588–0.736	< 0.001
Combination	0.87	0.77–0.98	0.022	0.583	0.513–0.663	< 0.001
PSA						
<10						
10–20	1.66	1.57–1.76	< 0.001	1.236	1.165–1.313	< 0.001
>20	3.07	2.87–3.28	< 0.001	1.592	1.481–1.71	< 0.001
Gleason						
≤ 6						
7	1.37	1.3–1.46	< 0.001	1.313	1.209–1.427	< 0.001
≥ 8	2.69	2.53–2.86	< 0.001	1.869	1.678–2.081	< 0.001

### Nomogram development for CSS and OS at 3,5 and 8 years

**Fig. 2:**
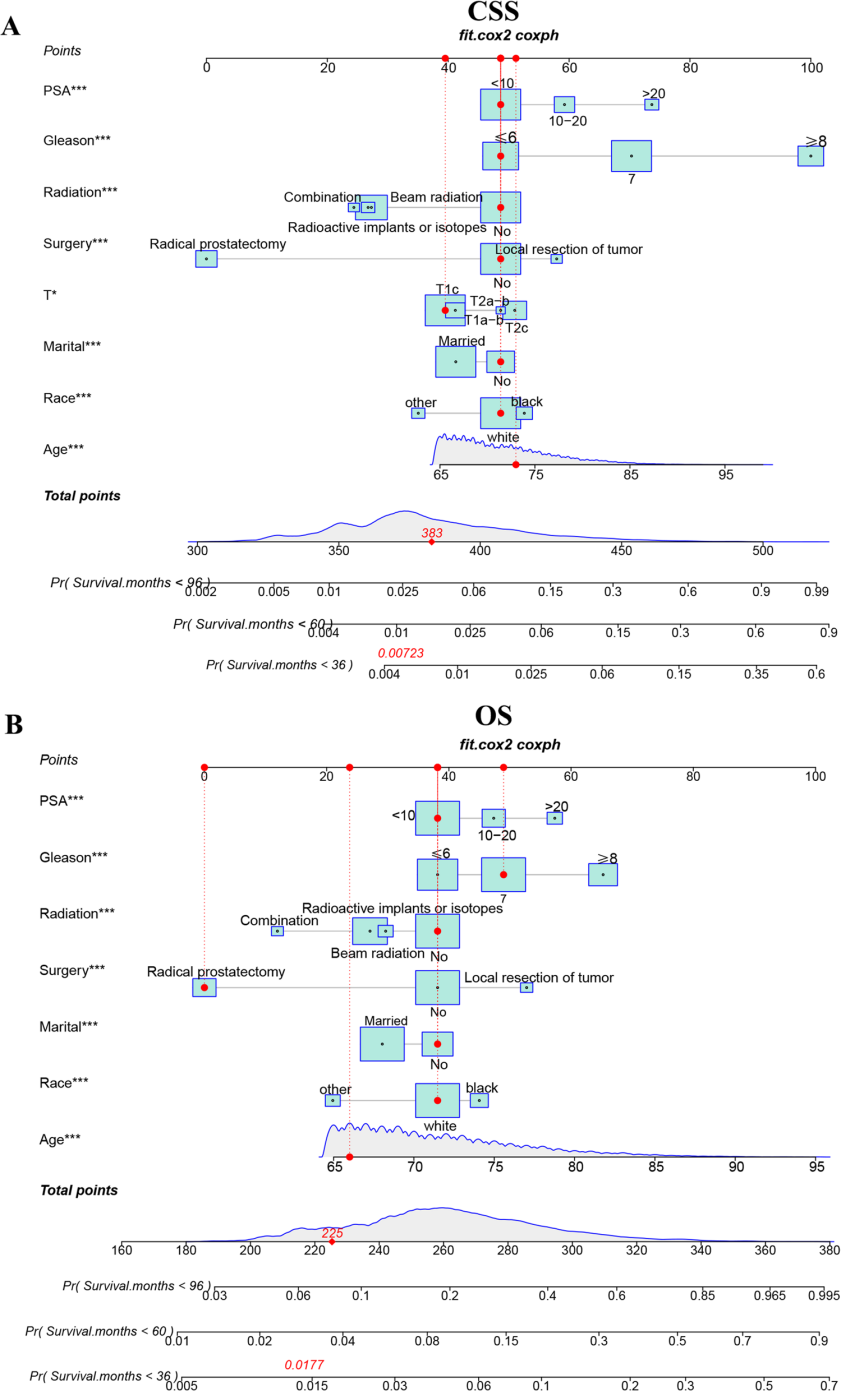
The nomograms for predicting 3-,5-,8-year CSS and OS in elderly patients with localized prostate cancer. **A** The nomogram for predicting CSS. **B** The nomogram for predicting OS

Based on the multivariable Cox regression model, we constructed new nomograms that could predict CSS and OS in elderly localized PC patients at 3,5 and 8 years (Fig. 2). The nomograms showed that surgery and radiotherapy were the most critical factors affecting CSS and OS in elderly patients with localized PC, followed by GS and PSA. In addition, age, marriage, and ethnicity influenced patient CSS and OS.

### Validation of the nomograms

**Fig. 3:**
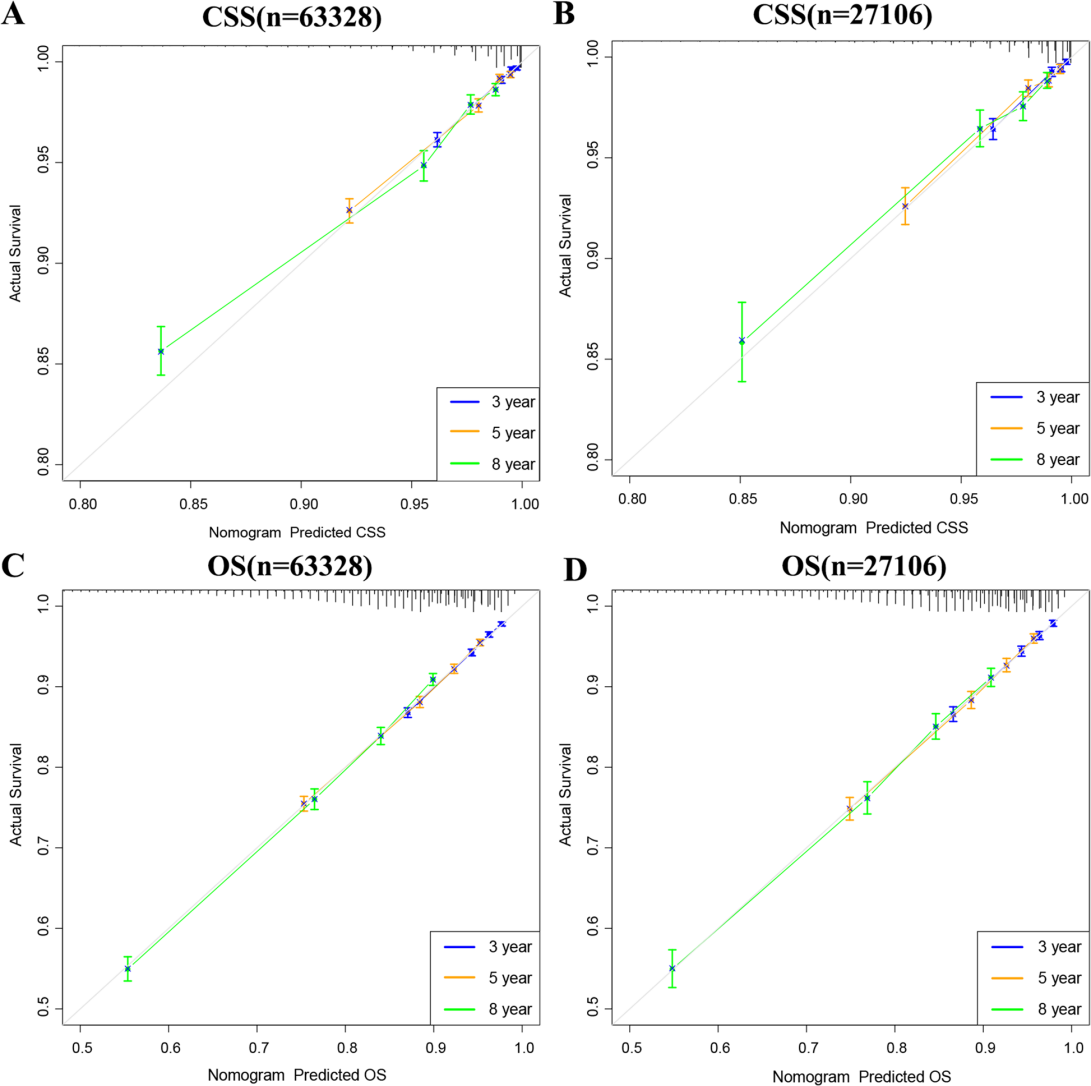
Calibration curve of the nomograms for predicting 3-,5-,8-year CSS and OS in elderly patients with localized prostate cancer. **A** Calibration curve of the nomograms for predicting CSS in the training set. **B** Calibration curve of the nomograms for predicting CSS in the validation set. **C** Calibration curve of the nomograms for predicting OS in the training set. **D** Calibration curve of the nomograms for predicting OS in the validation set. The horizontal axis is the predicted value in the nomogram, and the vertical axis is the observed value

**Fig. 4:**
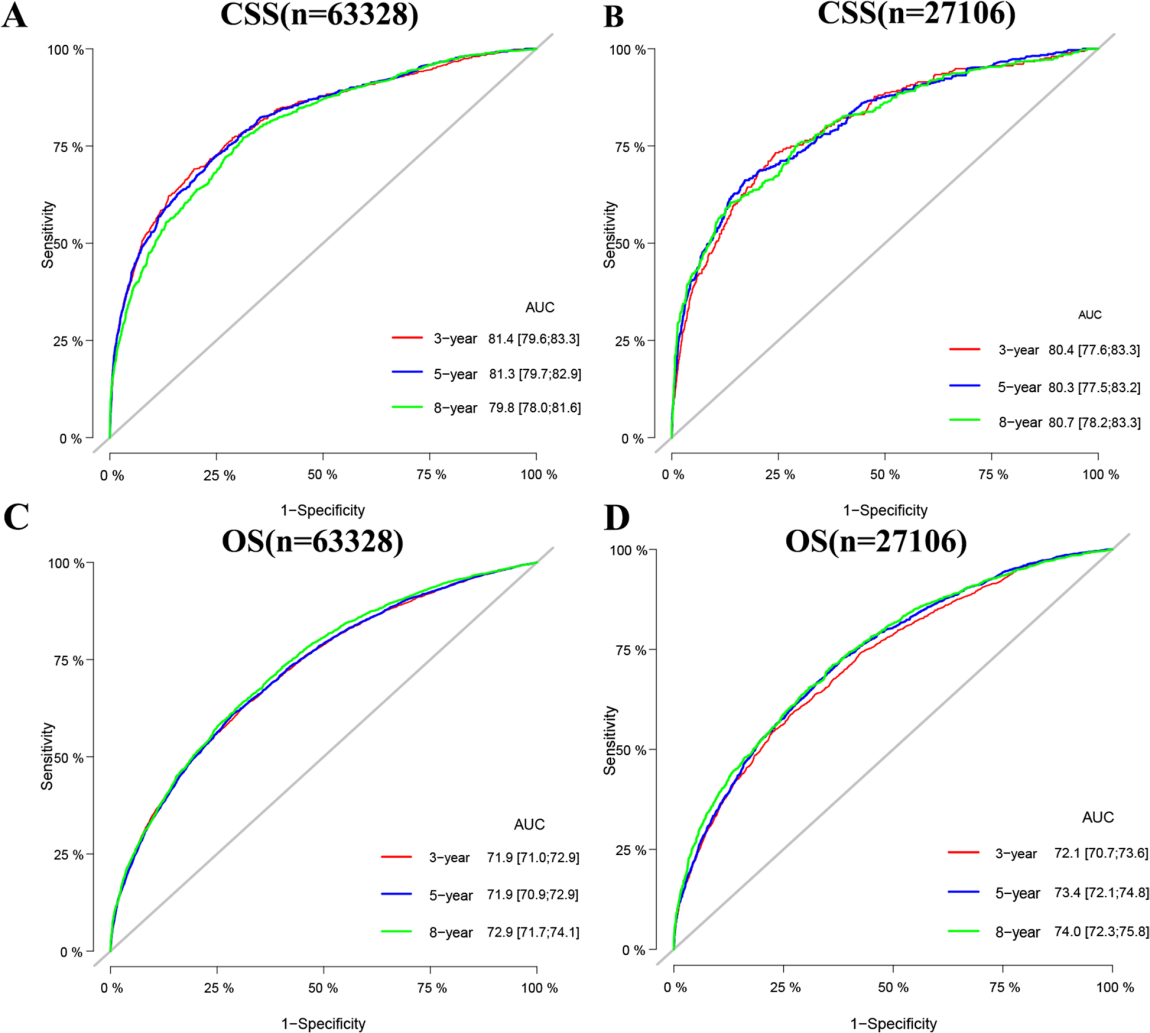
AUC for predicting 3-, 5-, and 8-year CSS and OS in elderly patients with localized prostate cancer. **A** The AUC at 3-, 5-, and 8-year for CSS in the training set. **B** The AUC for CSS in the validation set. **C** The AUC at 3-, 5- and 8-year for OS in the training set. **D** The AUC at 3-, 5- and 8-year for OS in the validation set

Internal cross-validation was used to test the accuracy and identifiability of the model. The c-index of the training and validation sets for the predicted CSS is 0.802(95%CI:0.788–0.816) and 0.798(95%CI:0.776–0.820, respectively). The predicted OS’s c-index of the training and validation sets is 0.712(95%:0.704–0.720) and 0.724(95%:0.714–0.734, respectively). The results show that the nomograms predicting CSS and OS have good discrimination power. The calibration curve shows that in the training and validation sets of the CSS and OS, the predicted values of this model are highly consistent with the actual observed values (Fig. 3). The results show that both CSS and OS nomograms have good accuracy. In the CSS training set, the AUC was 81.4,81.3, and 79.8, respectively, and in the CSS validation set, 80.4,80.3, and 80.7, respectively. In the OS training set, the AUC was 71.9,71.9, and 72.9, respectively, and in the OS validation set, the AUC was 72.1,73.4, and 74.0, respectively. The results showed that the nomogram has a strong discriminability (Fig. 4).

Furthermore, we performed external temporal validation, which shows that the external validation set C index for predicting CSS is 0.795(95%CI:0.764–0.826), while the external validation set C index for predicting OS is 0.728(95%CI:0.712–0.744). The calibration curve shows that in the external validation set of CSS and OS, the predicted values and the actual observations are highly consistent (Fig. S1). The AUC of the external validation set showed CSS of 79.2,77.3, and 78.1 at 1, 2, and 3 years, respectively, versus 72.5,69.9 and 69.9 at 1,2 and 3-year OS (Fig. S2).

### Clinical application of the nomograms

**Fig. 5:**
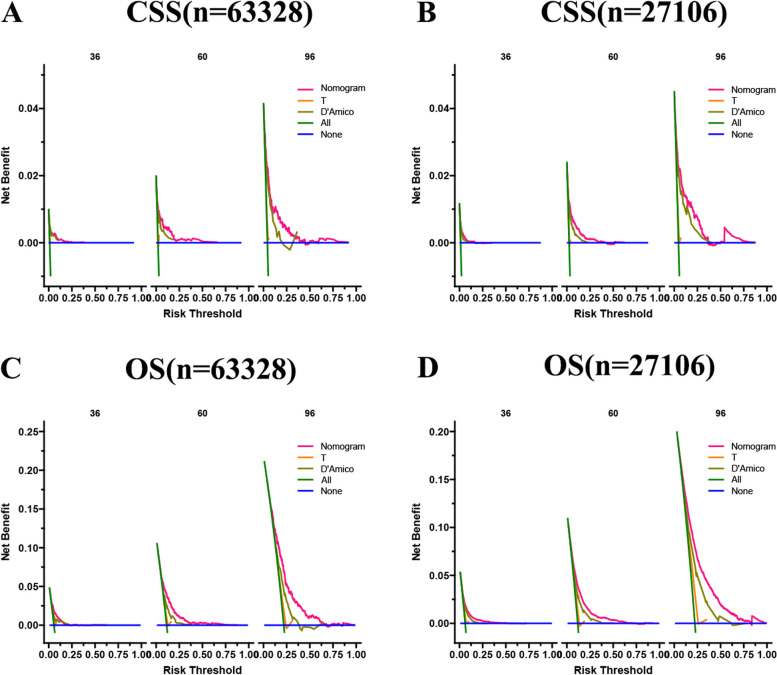
DCA of the nomograms for predicting CSS and OS. **A** The nomogram for CSS in the training set. **B** The nomogram for CSS in the validation set. **C** The nomogram for OS in the training and training set. **D** The nomogram for OS in the training and validation set

**Fig. 6:**
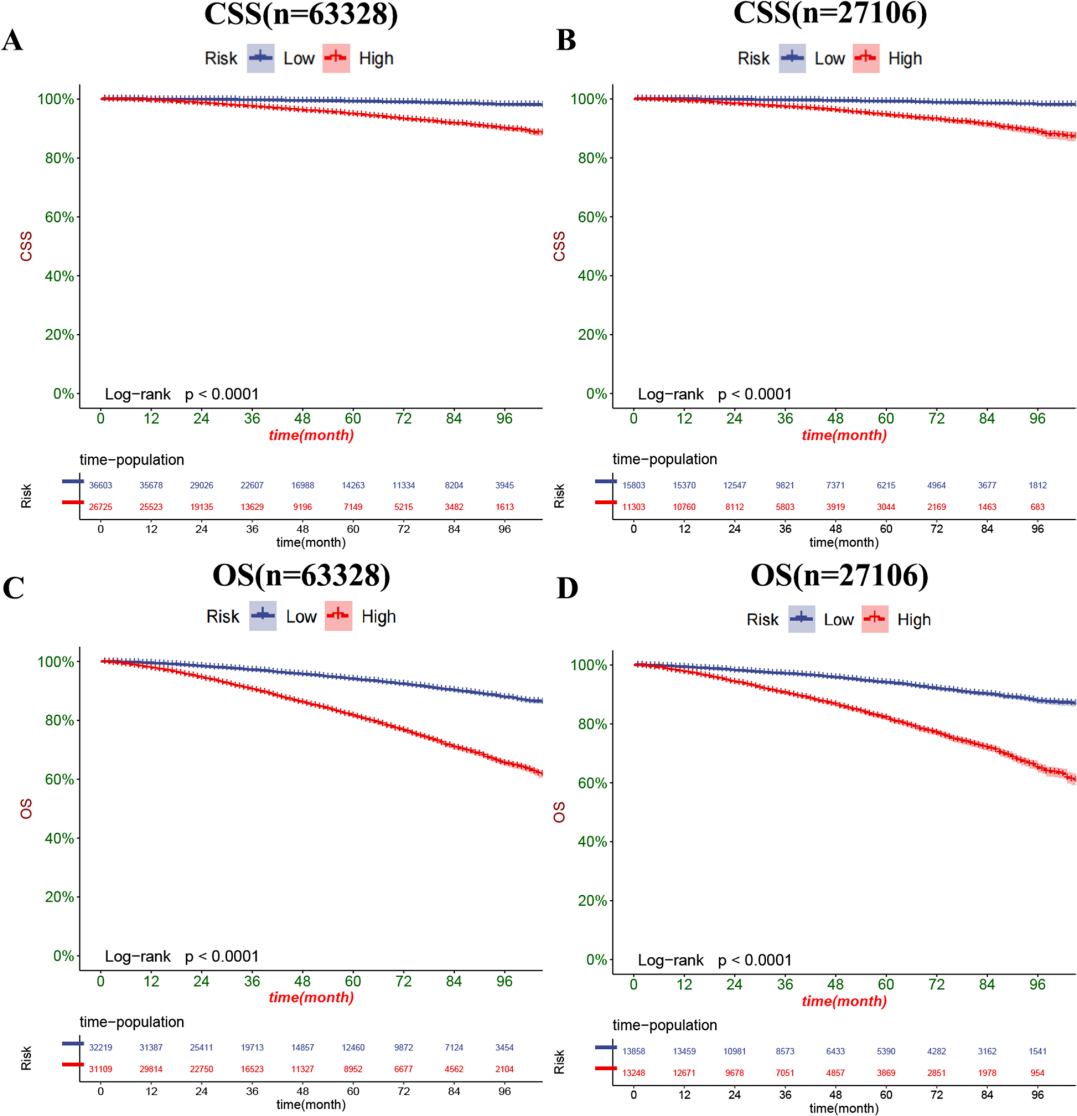
Kaplan-Meier curves of patients in the low-risk and high-risk groups. The K-M curve showed that the CSS rate of the patients in the high-risk group was significantly lower than that in the low-risk group both in the training set (**A**) and validation set (**B**). The K-M curve showed that the OS rate of the patients in the high-risk group was significantly lower than that in the low-risk group both in the training set (**C**) and validation set (**D**)

**Fig. 7:**
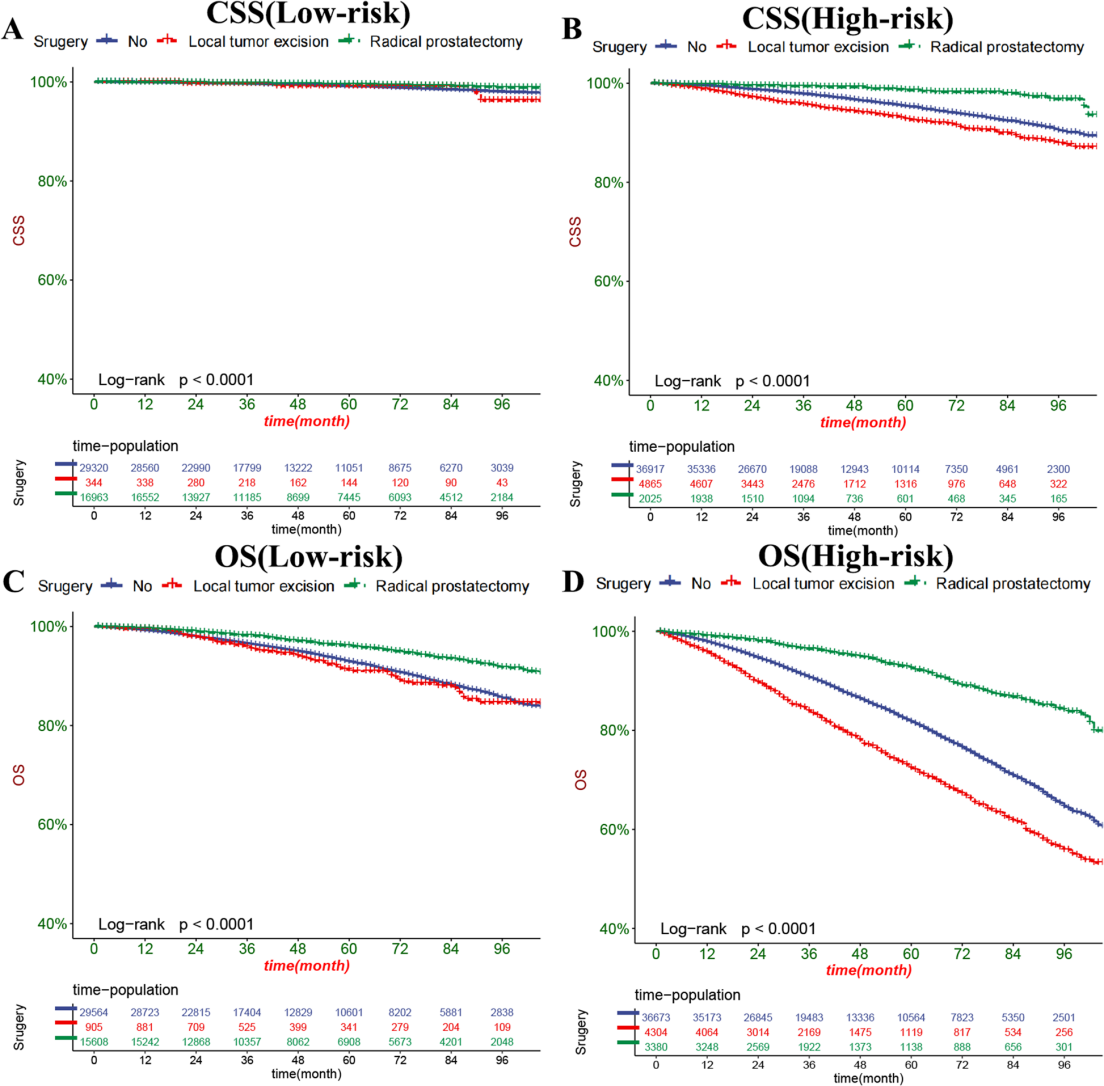
Kaplan-Meier curves of patients with surgery. **A** The CSS rate of patients in the low-risk group underwent different surgery. **B** The CSS rate of patients in the high-risk group underwent different surgery. **C** The OS rate of patients in the low-risk group underwent different surgery. **D** The OS rate of patients in the high-risk group underwent different surgery

The DCA results showed that our nomogram had good clinical potential value in both CSS and OS training and validation sets (Fig. 5). In the validation set of 3,5, and 8 years, both CSS and OS nomograms showed the best clinical potential value, followed by D’Amico risk stratification and the final T stage. We used the ROC curve to calculate the risk value and the optimal cutoff value for each patient. Patients were then divided into a high-risk group (total score of 180.23) and a low-risk group (total score < 180.23), a high-risk group with predicted OS (total score of 70.84), and a low-risk group (total score < 70.84). The K-M curve showed that CSS and OS rates were higher in low-risk than high-risk groups in both the training and validation set (Fig. 6). CSS rates at 3,5 and 8 years in the low-risk group were 99.7, 99.3, and 98.3%, respectively, while 3,5, and 8-year CSS rates were 97.6, 95.3, and 90.5% in the high-risk group. The 3-year, 5-year and 8-year survival rates were 97.1, 94.0, and 87.5%, respectively, compared with the 3-year, 5-year and 8-year OS rates of 90.5, 81.5, and 65.3% in the high-risk group. We found that in the low-and high-risk groups, patients had the highest rates of CSS and OS for radical prostatectomy, but most patients did not undergo surgery, with the lowest CSS and OS rates for local tumour resection (Fig. 7). However, the survival difference analysis of radiotherapy mode showed that patients in the high-risk group, patients who did not receive radiotherapy, had the lowest CSS and OS rates.

**Fig. 8:**
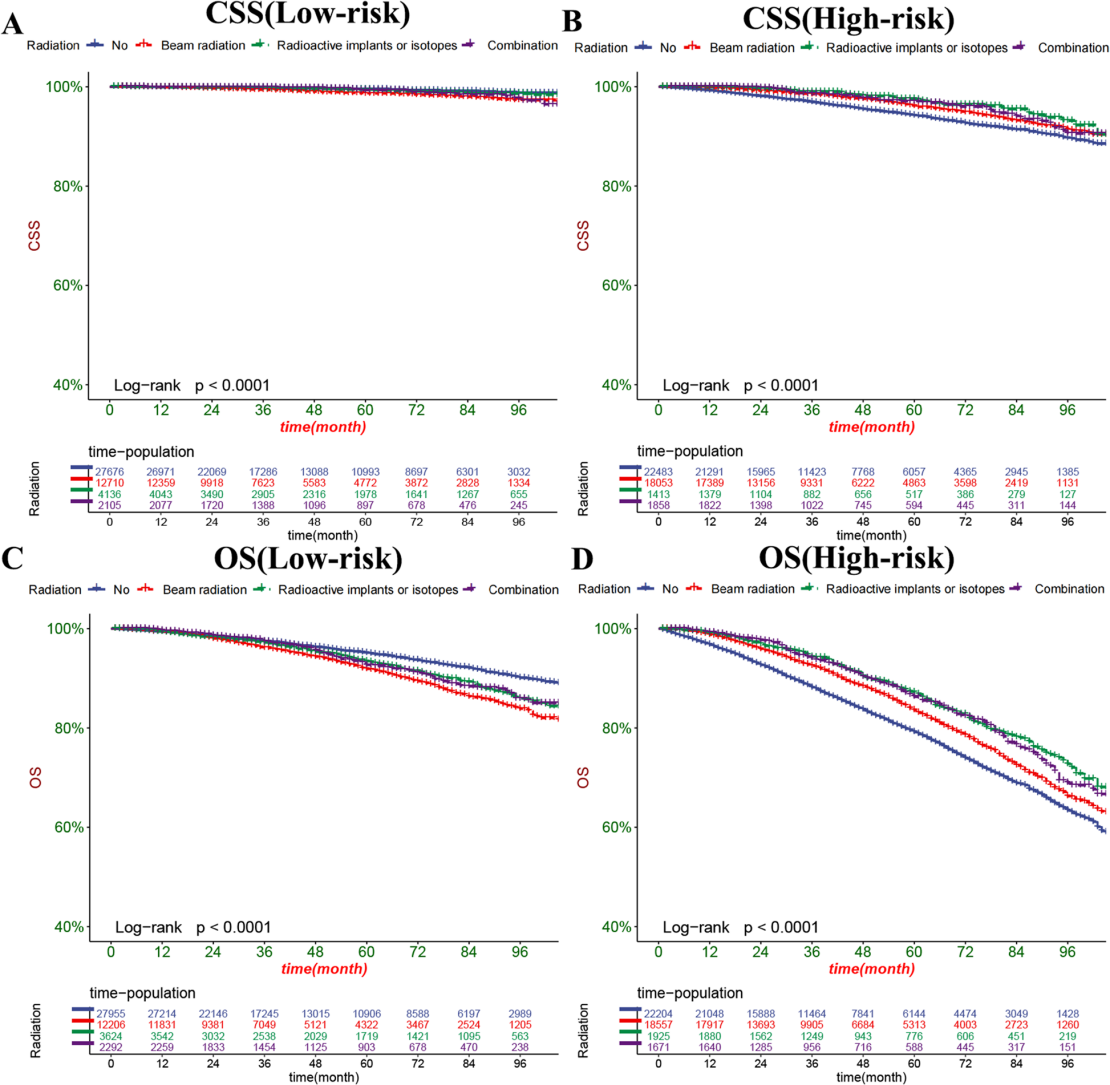
Kaplan-Meier curves of patients with radiotherapy. **A** The CSS rate of patients in the low-risk group underwent different radiotherapy. **B** The CSS rate of patients in the high-risk group underwent different radiotherapy. **C**: The OS rate of patients in the low-risk group underwent different radiotherapy. **D** The OS rate of patients in the high-risk group underwent different radiotherapy

In contrast, patients underwent CSS and OS rates with combined radiotherapy and Radioactive implants or isotopes, but both were higher than local irradiation. In the low-risk group, patients who did not receive radiotherapy had the highest CSS and OS rates. In contrast, local irradiation patients had the lowest CSS and OS rates. There was no significant difference in CSS and OS rates between patients combined with radiotherapy and Radioactive implants or isotopes (Fig. 8).

## Discussion

Localized PCs account for more than 80% of elderly patients [4]. However, due to the heterogeneity of the disease, it is imperative to find factors related to its prognosis, and we have a nomogram for predicting CSS and OS based on the SEER database.

The treatment of localized PC mainly focuses on the choice of active monitoring, surgery, and radiotherapy, and it is controversial whether localized PC is overtreated. A randomized controlled trial conducted by Anna Bill-Axelson et al. showed that radical prostatectomy reduced mortality and risk of metastasis in patients with localized PC [13]. Anna Bill-Axelson et al. also demonstrated a significant reduction in mortality after radical prostatectomy after long-term follow-up. In contrast, most long-term survivors in the observation waiting group did not need palliative care [14]. However, one study found that patients with localized PC undergoing local tumour resection were more likely to relapse than those treated with radical resection [15]. Our nomogram showed that surgery is the most critical factor in predicting CSS and OS in elderly patients with localized PC, consistent with previous studies. At the same time, the KM curve for the surgical method showed that the best survival rate could be obtained for all the patients with radical prostatectomy.

In contrast, the patients with local tumour resection had the lowest CSS and OS rates at 3,5 and 8 years, considering that the reason may be that fewer patients with local tumour resection could lead to the resulting bias. Secondly, most patients with localized PC only need active monitoring to obtain a better prognosis, so most patients do not receive surgical treatment. Their survival rate is not lower than that of patients undergoing local tumour resection. Based on this, radical prostatectomy is recommended for elderly patients with localized PC if conditions permit. Secondly, active surveillance is a better option for patients who cannot tolerate surgery.

RT provides treatment for localized PC without major surgery and is the preferred treatment for many men [16]. Nina Samson et al. showed that reduced doses of EBRT appeared to be a viable alternative to ADT in older patients with localized PC [17]. Hiromichi Ishiyama et al. confirmed that combination radiotherapy is a safe and effective treatment for patients with localized PC [18]. While our nomogram also found that radiotherapy was another critical factor in predicting CSS and OS in elderly patients with localized PC, radiotherapy was associated with a better prognosis, consistent with previous studies. However, the KM curve for radiotherapy methods showed that patients in the high-risk group had the highest CSS and OS at 3,5 and 8 years, while patients in the low-risk group had the highest survival rate without receiving radiotherapy. The reason may be that low-risk localized PC patients can obtain a better prognosis through active monitoring.

In contrast, elderly patients cannot tolerate the side effects of radiotherapy due to comorbidities, so receiving radiotherapy leads to a worse prognosis. We do not recommend the routine use of radiotherapy for low-risk elderly patients with localized PC. However, radiotherapy is recommended for elderly patients with a high risk.

Chemotherapy is not the preferred treatment for PC, and previous studies also showed no significant prognostic benefit in PC [19]. The conventional TNM stage is strongly related to PC survival. Frederik B Thomsen et al. also showed a higher T stage, a worse prognosis for PC patients, and a higher risk of metastasis [20]. However, our multivariate COX regression analysis showed that neither T stage nor chemotherapy was an independent risk factor for predicting OS. While chemotherapy is a risk factor but not an independent risk factor for CSS in elderly localized PC patients, the reason may be high non-cancer-specific mortality in elderly patients due to comorbidities. Hence, T-stage and chemotherapy are not independent risk factors affecting OS. Secondly, many elderly patients cannot tolerate the toxic side effects of chemotherapy, which makes the prognosis of patients receiving chemotherapy worse, making chemotherapy have no benefit for CSS. The small number of people who finally received chemotherapy has biased the results. However, the nomogram of CSS showed that the prognostic difference in the T stage was not very significant, considering that the T stage of localized PC patients was limited to the T1 and T2 stages, so the overall prognosis was good.

GS score and PSA level are crucial for the prognosis of PC patients, and our study selected GS grading criteria and PSA grading criteria using the criteria in D’Amico risk stratification [21]. Our nomogram showed that GS and PSA were also independent risk factors for predicting CSS and OS in patients with localized PC and that higher GS and PSA were associated with worse outcomes, consistent with clinical experience [22]. The data showed that 20-year PC patients with 2–6,7 and 8–10 cancer-specific mortality were 10, 4%0 and 70%, respectively [23]; previous nomograms also showed that PSA is a critical factor in PC outcomes [24], consistent with our conclusion.

Age is closely related to the incidence of PC, and our nomogram shows that age is an independent risk factor for CSS and OS in elderly localized PC patients. Most previous prediction models for PC also revealed that age is a risk factor for PC patients, and older patients are associated with a poor prognosis [25–27]. Secondly, marital status and ethnicity were independent factors affecting CSS and OS in elderly patients with localized PC. Our nomogram shows marriage as a protective factor, consistent with previous studies [28]. Cancer patients receive more financial support and psychological comfort through marriage, and married patients seek treatment more actively out of responsibility to their families [29, 30]. Race is also an independent risk factor for CSS and OS in patients with localized PC. Our nomogram showed that blacks were associated with a worse prognosis, followed by white people and other races, consistent with the conclusions of previous studies [31, 32].

This study explored the risk factors for CSS and OS in elderly patients with localized PC, where we developed nomograms to assist clinicians and patients in assisted decision-making. However, studies based on the SEER database have limitations, starting with the need for more active surveillance and ADT-related data. These two are the preferred measures for treating many PC patients. Active monitoring can achieve a better prognosis, especially for many patients with localized PC. Second, this study was retrospective, and it is challenging to avoid selection bias. Finally, our study lacks critical information such as specific chemotherapy regimen, dose and course of treatment. However, this study still included many key clinicopathological factors and underwent internal and temporal external validation so the results would not be too biased.

## Conclusions

We explored the influencing factors of CSS and OS in elderly patients with localized PC, and we found that surgery, radiotherapy, T stage, GS, PSA, age, race, and marriage were independent risk factors for CSS; however, T stage was not an independent risk factor for OS. We developed new nomograms to predict CSS and OS in elderly localized PC patients. With internal and external validation, models showed good accuracy and reliability. DCA showed that our nomogram could guide clinicians and patients to make better decisions due to the T staging and D’Amico risk stratification systems.

### Supplementary Information

**Additional file 1: Figure S1.** Calibration curve in the external verification of CSS and OS in elderly patients with localized prostate cancer. **Figure S2.** The AUC in the external verification of CSS and OS in elderly patients with localized prostate cancer.

## Data Availability

The SEER data analyzed in this study is available at https://seer.Cancer.gov/.
