# Supplementary figures and images for: Prognostic significance of surgery and radiotherapy in elderly patients with localized prostate cancer: establishing and time-based external validation a nomogram from SEER-based study
Source: BMC Urol. 2024 Jan 6;24:12. doi: 10.1186/s12894-023-01384-6 (PMC10771675; doi:10.1186/s12894-023-01384-6)

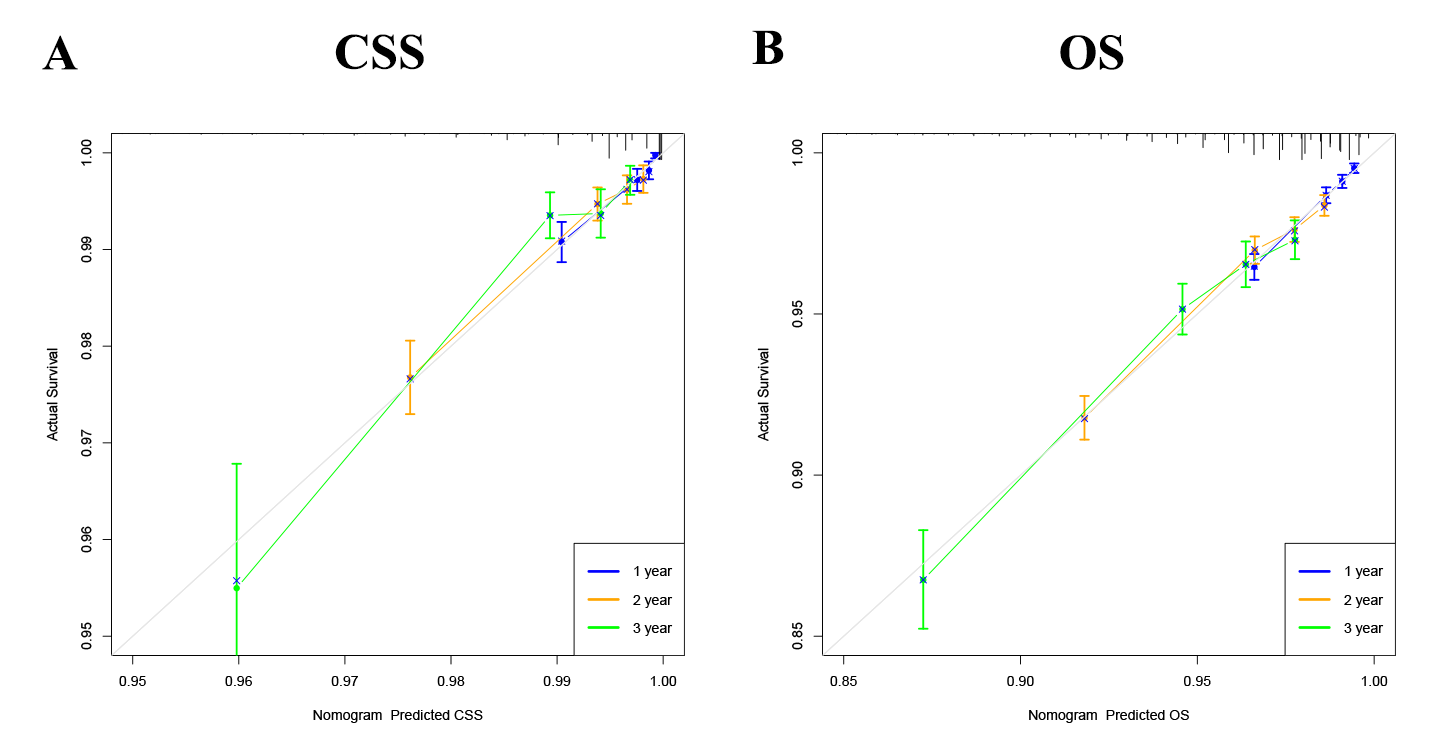

Supplement: Supplementary file 1 — Additional file 1: Figure S1. Calibration curve in the external verification of CSS and OS in elderly patients with localized prostate cancer. Figure S2. The AUC in the external verification of CSS and OS in elderly patients with localized prostate cancer. [file 12894_2023_1384_MOESM1_ESM.zip › 12894_2023_1384_MOESM1_ESM.tif]

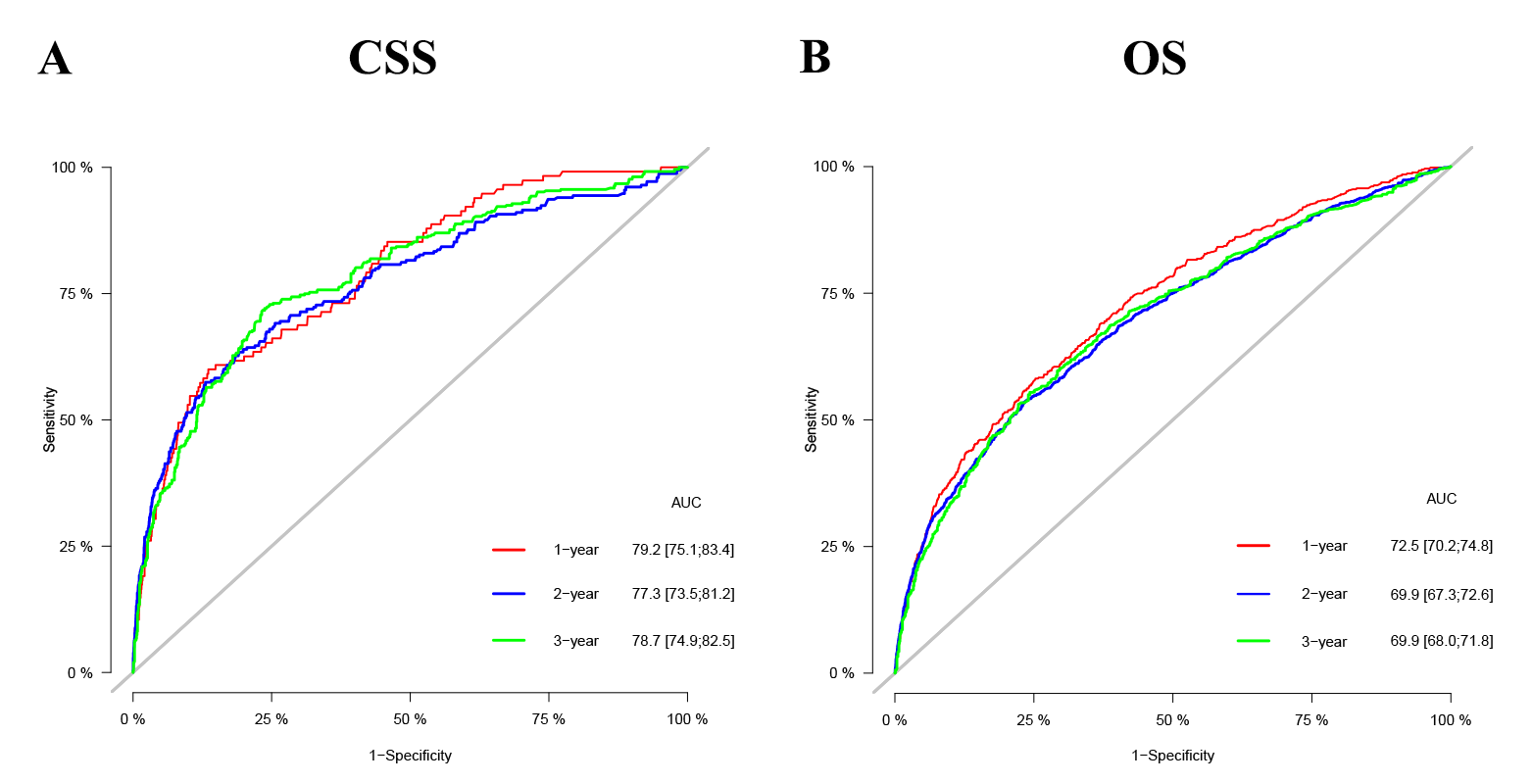

Supplement: Supplementary file 1 — Additional file 1: Figure S1. Calibration curve in the external verification of CSS and OS in elderly patients with localized prostate cancer. Figure S2. The AUC in the external verification of CSS and OS in elderly patients with localized prostate cancer. [file 12894_2023_1384_MOESM1_ESM.zip › 12894_2023_1384_MOESM2_ESM.tif]
